# Attempt of Bayesian Estimation from Left-censored Data Using the Markov Chain Monte Carlo Method: Exploring Cr(VI) Concentrations in Mineral Water Products

**DOI:** 10.14252/foodsafetyfscj.D-20-00007

**Published:** 2020-12-25

**Authors:** Yoshinari Suzuki, Noriko Tanaka, Hiroshi Akiyama

**Affiliations:** 1 Division of Foods, National Institute of Health Science, Tonomachi 3-25-26, Kawasaki-ku, Kawasaki, Kanagawa 210-9501, Japan; 2 Department of Health Data Science Research, Healthy Aging Innovation Center, Tokyo Metropolitan Geriatric Medical Center, Sakae-cho 35-2, Itabashi-ku, Tokyo 173-0015, Japan

**Keywords:** nondetects, left-censored data, Bayesian model, MCMC, Stan, Cr(VI)

## Abstract

Hexavalent chromium (Cr(VI)) is toxic, carcinogenic, and mutagenic substances. Oral exposure to Cr(VI) is thought to be primarily from drinking water. However, under the certain reporting limit (~0.1 µg/L), percentage of Cr(VI) concentration in mineral water products under the reporting limit were estimated higher than 50%. Data whose values are below certain limits and thus cannot be accurately determined are known as left-censored. The high censored percentage leads to estimation of Cr(VI) exposure uncertain. It is well known that conventional substitution method often used in food analytical science cause severe bias. To estimate appropriate summary statistics on Cr(VI) concentration in mineral water products, parameter estimation using the Markov chain Monte Carlo (MCMC) method under assumption of a lognormal distribution was performed. Stan, a probabilistic programming language, was used for MCMC. We evaluated the accuracy, coverage probability, and reliability of estimates with MCMC by comparison with other estimation methods (discard nondetects, substituting half of reporting limit, Kaplan-Meier, regression on order statistics, and maximum likelihood estimation) using 1000 randomly generated data subsets (*n *= 150) with the obtained parameters. The evaluation shows that MCMC is the best estimation method in this context with greater accuracy, coverage probability, and reliability over a censored percentage of 10-90%. The mean concentration, which was estimated with MCMC, was 0.289×10^−3^ mg/L and this value was sufficiently lower than the regulated value of 0.05 mg/L stipulated by the Food Sanitation Act.

## 1. Introduction

Chromium (Cr) is a metal widely distributed in the environment. Chromium has various oxidation states from −2 to +6, but the most predominant forms are +3 and +6^1^^)^. Trivalent Cr (Cr(III)) is an essential nutrient for cholesterol, fat and glucose metabolism in human bodies, while hexavalent Cr (Cr(VI)) is toxic, carcinogenic, mutagenic, and mobile in nature^2^^)^. Continuous exposure to Cr(VI) may cause pulmonary congestions, liver damage, skin irritation, and kidney failure^3^^)^. Once Cr(VI) enters inside the cell, it undergoes a rapid metabolic reduction and is converted ultimately to Cr(III)^4^^)^. Thus, the Food Safety Commission of Japan estimated daily intake of Cr(VI) from consumption of mineral water and tap water^5^^)^. According to the reports on Cr(VI) concentration in mineral water (MW) products^6^^,^^7^^,^^8^^)^, detected concentrations ranged from 0.1 to 3.4 µg/L, and the data contained many non-detected values with censored percentage from 50 to 75% under the certain reporting limit (~0.1 µg/L). When nondetects are present in data, they lead to difficulties in computing statistical metrics. Estimates from the data with nondetects could vary widely depends upon not only the censored proportion due to the properties of the measured substance and the performance of the analytical instrument, but also the performance and the assumption of the statistical analysis methods. In other words, estimation of Cr(VI) exposure via MW products becomes uncertain.

Since foods are major exposure source to chemicals, assessment of chemical exposure via foods is important for health protection. Non-detected values often occur for chemical concentrations in foods because the analytical methods for chemicals in foods always have the limit of detection (LOD) and the limit of quantification (LOQ)^9^^,^^10^^,^^11^^,^^12^^,^^13^^)^. Data lacking certain values which are lower ​​than LOD and LOQ, are known as left-censored. In the field of food analytical science, many studies used substitution (0^10^^,^^11^^)^, LOD/2^10^^,^^13^^)^, or LOD^9^^,^^12^^)^) for left-censored data on the exposure estimation. The estimation of chemical exposure via foods is likely to be evaluated by the conservative method using substitution of LOD or LOQ for left-censored data on the safety side. Therefore, such conservative assessment using those methods may cause a severe overestimation that is meaningless and impractical from the viewpoint of health risk and food distribution. False concerns and excessive regulations lead to strict rules onto the society and the food community. In 2006, EPA guidance supported the use of 0, LOD/2, or LOD substitution in data with less than 15% nondetects^14^^)^. However, in 2015 the EPA revised its guidelines stating that substitution by LOD/2 should be used only if the percentage of censored data is less than 5% and if the data are mildly skewed^15^^)^. Helsel^16^^)^ advocates that academic journals should consider substitution as flawed and reject the papers that such methods are implemented. Helsel recommended three methods—Kaplan-Meier (KM), maximum likelihood estimation (MLE), and regression on order statistics (ROS)—as more accurate alternatives for computing statistics on data with nondetects^17^^,^^18^^)^. Helsel^18^^)^ gave recommendations of usage of KM, MLE, and ROS methods as follows: 1: KM should be used for data with censored percentage of <50%; 2: ROS should be used for small sample size (<50) and 50-80% censored percentage; 3: MLE should be used for large sample size (≥50) and 50-80% censored percentage. It can be expected that the MLE shows good performance for large sample size, however it is also reported that MLE shows poor performance for high-skewed data^19^^)^. On the contrary, it is well known that incorporating prior information in Bayesian framework result in a reduced sample size, and Bayesian method allows for great flexibility in dealing with missing data^20^^)^. Thus, we expect that Bayesian framework may give a unified solution for different situations of left-censored data.

In singular models with many hidden parameters, such as multinomial mixture model, change-point model, neural networks, hidden Markov models, and so on, it was reported that Bayesian estimation make the generalization error smaller^21^^)^. Since left-censored data have many hidden values as nondetects, Bayesian estimation is expected to provide appropriate estimates. Thus, the estimation method based on Bayesian modeling using the Markov chain Monte Carlo (MCMC) method has recently been applied in left-censored data. To compute Bayesian models, probabilistic programing languages such as BUGS and JAGS have been used. For example, Bayesian modeling using BUGS and JAGS has been applied to left-censored data for microbiological contamination data^22^^)^, hexabromocyclododecane diastereomer compositions in water^23^^)^, fluoride in drinking water^24^^)^, and so on.

While there are various reports on comparisons among non-Bayesian methods (substitution, non-parametric, semi-parametric, and parametric) in the context of left-censored data analysis^25^^,^^26^^,^^27^^,^^28^^,^^29^^,^^30^^)^, the reports comparing the performance of Bayesian estimation with other estimation methods are limited. Huynh et al^31^^)^ evaluated the estimation performance of a Bayesian method and substitution by simulation with real data in the specific area except for food safety science. Nie et al^32^^)^, Hady and Rain^33^^)^, and Feroze and Aslam^34^^)^ also evaluated by comparison of Bayesian and likelihood-based estimation for left-censored data using simulations. Although Nie el al^32^^)^ adopted Bayesian approach to the real data in the specific area except for food safety science, Hady and Rain^33^^)^, and Feroze and Aslam^34^^)^ performed a simulation study without real data. They revealed that Bayesian method resulted in less bias. Although Bayesian estimation has been reported to have satisfactory coverage probability, as well as less bias, the comparable performance and relative reliability (i.e., the stability of results given alternative datasets) of the Bayesian method approach in food safety science have been never evaluated. A method with less reliability may fail to fit additional data or predict future observations. We consider that it is important to validate the Bayesian method approach for the standardization in food safety science.

In this study, in order to elaborate exposure assessment, we applied Bayesian modeling using MCMC, in addition to non-Bayesian methods, to obtain less biased summary statistics on Cr(VI) concentrations in MW products from Kataoka et al (2017)^8^^)^, which has over 50% nondetects and no specified criterion concerning nondetects. Since the true values of probability density distribution parameter are unknown, we have conducted a simulation study. In addition, we validated the proposed MCMC method on the left-censored data analysis by comparison of several estimation methods to explore the accuracy, coverage probability, and reliability of the proposed MCMC method in food safety science. The process was divided into two stages as follows: 1: Estimate the concentration distribution from the original data using MCMC and other methods; 2: Evaluate MCMC performance compared with other statistical methods from simulation study. Finally, this study can contribute to the reliability of food safety evaluation by indicating the possibility of evaluating more accurate concentration in a similar situation to Cr(VI).

## 2. Methods

### 2.1 Data

Data on Cr(VI) concentrations in MW products reported by Kataoka et al^8^^)^ were used. One hundred and fifty MW products were purchased in 2016. Among them, 110 products are domestic, and 40 products are imported. The number of purchases was assigned according to the number of products for each prefecture and country in the Japanese market. The lower LOQ was calculated and data below LOQ are marked as “Tr.”. However, no description about the value of LOQ can be found in the text. Although summary statistics for the observed data are reported in the paper, appropriate analysis for data containing nondetects has not been performed. Herein, unless otherwise specified, the censoring criterion is described as reporting limit (RL).

### 2.2 Inferential Analysis

Statistical analysis was performed using R (ver. 3.4.0). R packages EnvStat (ver. 2.3.1) and rstan (ver. 2.16.2) were used to estimate parameters from left-censored data.

### 2.3 Parameter Estimation

To compare parameters estimated from left-censored data with MCMC, we used 5 other methods (1: Discard nondetects (DN); 2: Substitution of one half of RL (RL/2); 3: KM; 4: MLE; 5: ROS). ROS is a method corresponding to a normal or lognormal distribution. We performed statistical analysis under assumption that the original data follows lognormal distribution (see **Supporting Information S4**. for further detail). KM, MLE, and ROS methods were implemented using the EnvStats package. The minimum value of detected data was used as a censoring point for convenience. Then a location parameter (geometric mean, $\hat{m}$), a shape parameter (geometric standard deviation, $\hat{s}$), a mean ($\hat{\mu }$), a standard deviation ($\hat{\sigma }$), and lower (LCI, $\hat{L}$) and upper (UCI, $\hat{U}$) 95% confidence intervals of mean Cr(VI) concentration were estimated for data reported by Kataoka et al^8^^)^ using the 6 methods.

#### 2.3.1 Discard nondetects

Entries with non-detected values are eliminated and only detected values are used for further calculation. This approach is attractive because of its simplicity but the data may be distorted.

#### 2.3.2 Substitution of one half of reporting limit

Non-detected values are replaced with one half of RL and both detected and replaced values are used for further calculation. Substitution is still widely used in various fields because of its tractability, and it is perhaps acceptable under certain conditions.

#### 2.3.3 Kaplan-Meier

Kaplan-Meier is a nonparametric estimator since it does not make distributional assumptions. More specifically, KM is a non-parametric version of maximum likelihood estimation, and this method estimates the percentiles, or cumulative distribution function (CDF), for the data, where the mean equals the area beneath the CDF^35^^)^. It is widely used in survival analysis to estimate survival functions, which are then used to estimate different summary statistics^35^^)^ (see **Supporting Information S6.1** for the detailed algorithm). The KM method can provide useful estimates when sample sizes are small; however, it does not perform well if more than 50% of data are nondetects or if fewer than eight detections are available for evaluation^18^^)^.

#### 2.3.4 Regression on order statistics

Regression on order statistics is a semi-parametric, simple imputation method that transforms nondetects on the basis of a probability plot of detects^17^^,^^18^^,^^36^^,^^37^^)^. ROS is also known as “Imputation Using Quantile-Quantile Regression” (see **Supporting Information S6.2** for the detailed algorithm). ROS performs better than MLE and some substitution methods when the sample size is small (less than 50) and where data do not fit a distribution^38^^)^.

#### 2.3.5 Maximum likelihood estimation

Maximum likelihood estimation solves a likelihood equation to estimate the parameter(s) using both detected observations and the proportion of data falling below RL^17^^)^. The observed data (*x*) enter the likelihood function through the probability density function (PDF, *f*(*x*|*m*, *s*)) of lognormal distribution and the censored observations can be accounted for by the CDF (*F*(RL|*m*,*s*) = *P*(*x* ≤ RL|*m,s*)) of lognormal distribution as follows:

$$L\left(m,s\text{|}x_{1},x_{2},\cdots ,x_{n}\right)=\underset{x\in D}{\prod }f\left(x\text{|}m,s\right)\cdot \underset{x\in C}{\prod }F\left(\text{RL|}m,s\right)\;,\;\cdots \;Eq.1$$

where *D* is the set of all observed values and *C* is the set of all left-censored values. MLE finds the parameter values (*m*, *s*) that maximize the likelihood function against the observations (see **Supporting Information S6.3** for the detailed algorithm).

#### 2.3.6 Markov chain Monte Carlo

Among probabilistic programing languages, Stan which was developed by Gelman et al^39^^)^ was used in this study. A key feature of Stan is that NUTS (No-U-Turn Sampler), which is an implementation of Hamiltonian Monte Carlo, one of the algorithms of the MCMC method, is adopted. Various Bayesian modeling sources using Stan have been published and documentation is abundantly available online^40^^)^.

In addition to two parameters of distribution (GM and GSD), we estimated RL with using MCMC (see **Supporting Information S1.** for furter details). Four parallel Markov chains are calculated using MCMC to check convergence. The numbers of iterations, warm-up, and thinning were set to 2×10^3^, 1×10^3^, and 2, respectively (see **Supporting Information S2.** for further details). For parameters estimated by MCMC, we used an expected a posteriori (EAP), which is mean of the posterior predictive distribution, as a point estimate. The reason is that an EAP reflect the information of entire distribution, while maximum a posteriori can be significantly affected by a small number of outliers. Then EAP was used for further calculation. After execution of the Stan model, we calculated the widely applicable information criterion (WAIC)^21^^)^ (see **Supporting Information S3.** for further details).

### 2.4 Monte Carlo Simulations

We generated 10^5^ random numbers according to the lognormal distribution with the parameters estimated in section 2.3. Using MCMC, estimated parameter(s) can be obtained as posterior predictive distribution(s). Since we obtained a set of 2×10^3^ estimates for each parameter, 50 random numbers are generated against posterior predictive distributions, and a total of 10^5^ random numbers are obtained (see line 42 of **Fig. S1**).

### 2.5 Simulation and Comparison of Various Methods

To explore the accuracy, coverage probability, and reliability of parameter estimation from left-censored data by MCMC, simulation and comparison with other methods (DN, RL/2, KM, ROS, and MLE) was carried out. To rank the 6 methods for estimating left-censored data, simulations were performed with the following algorithm:

1. Generate a random number of size 150 according to a lognormal distribution with the estimated parameters in section 2.3.6 as true values.

2. Simulate censoring by using a particular theoretical percentile of the sample data as an RL.

3. Execute the various methods to estimate parameters.

4. Repeat steps 1-3 1000 times.

5. Using the true values, compute some criteria.

When simulating censoring, we used theoretical percentiles of simulated data. For example, to simulate data with 40% nondetects, we considered any value falling below the theoretical 40^th^ percentile of the data as nondetects. We considered the minimum value of detected data as RL. The simulations cover multiple censored percentages from 10 to 90%. Then 6 parameters ($\hat{m}$ , $\hat{s}$ , $\hat{\mu }$ , $\hat{\sigma }$ , $\hat{L},$ , and $\hat{U}$) were estimated using the 6 methods.

To evaluate accuracy, bias and mean squared error (MSE) were calculated. The bias of an estimator $\hat{\theta }$, for estimating parameter θ, is defined as follows:

$$B\left(\hat{\theta }\right)=E\left(\hat{\theta }-\theta \right)\;.\;\cdots \;Eq.2$$

MSE of an estimator $\hat{\theta }$ is defined as follows:

$$\text{MSE}\left(\hat{\theta }\right)=E\left[{\left(\hat{\theta }-\theta \right)}^{2}\right]\;.\;\cdots \;Eq.3$$

We calculated the coverage probability of 95% CIs for mean (CP), which indicates the fraction of computed CIs that include the desired but unobservable parameter value. The 95% CIs for means estimated by DN and RL/2 were calculated using Cox’s method^41^^)^ (see **Supporting Information S5.** for further details). The 95% CIs for means estimated by KM, ROS, and MLE were calculated by the bootstrap with bias-corrected and accelerated method (with 1000 repetitions). For the MCMC method, 95% credible intervals (CrI) for the mean were calculated (see line 43 of **Fig. S1**). Then the CP was evaluated by calculating whether the expected value was included within the 95% CI or 95% CrI (see **Supporting Information S7.** for further information of CI and CrI).

To evaluate reliability, which indicates the stability of results given alternative data, we calculated the relative interquartile range (RIQR) and relative standard deviation (RSD) for estimated parameters of GM and GSD from 1000 different datasets.

## 3. Results

### 3.1 Method Inter-comparison for Cr(VI) concentrations with Nondetects

To validate the MCMC results, we conducted a method inter-comparison. Table 1 shows summary statistics and estimated parameters from left-censored data by various methods. The following describes the results from each estimation method.

**Table 1.** Summary statistics and estimated parameters from left-censored data based on six inferential methods.

Method	Mean (×10^−3^)	SD (×10^−3^)	LCI^b^ (×10^−3^)	UCI^b^ (×10^−3^)	GM (×10^−3^)	GSD
DN	0.472	0.433	0.380	0.587	0.348	2.18
RL/2	0.233	0.353	0.187	0.291	0.114	2.97
KM	0.261	0.338	0.216	0.305	0.160	2.69
ROS	0.257	0.343	0.208	0.398	0.148	2.78
MLE	0.266	0.846	0.201	0.342	0.080	4.72
MCMC	0.289^a^	1.06 ^a^	0.194	0.460	0.082^c^	4.90^c^

a: Expected a posteriori of generated quantities (mean_est or sd_est) calculated by MCMC.

b: Lower and upper 95% confidence interval of the mean. For MCMC, 95% credible interval of posterior predictive distribution for generated quantities (mean_est).

c: Expected a posteriori of target parameters (GM and GSD).

#### 3.1.1 Discard nondetects

Among the estimation methods, ${\hat{\mu }}_{\;}^{\text{DN}}$ (0.472×10^−3^) and ${\hat{s}}_{\;}^{\text{DN}}$(2.18) were highest and lowest, respectively. Given that nondetects are ignored, it seems reasonable to have such a result.

#### 3.1.2 Substitution of one half of reporting limit

Although the 95% CI of ${\hat{\mu }}_{\;}^{\text{RL}/2}$ overlapped with that estimated by other methods except for DN, ${\hat{\mu }}_{\;}^{\text{RL}/2}$ (0.233×10^−3^) showed the minimum value among the estimation methods. Since the dataset used in this study does not satisfy the EPA’s proposed requirements for RL/2^15^^)^, we should not use the RL/2 method.

#### 3.1.3 Kaplan-Meier

If there is only one RL, the estimation result is that ${\hat{\mu }}_{\;}^{\text{KM}}$ is identical to a substitution of the RL for censored data^18^^)^. Thus, it is reasonable that ${\hat{\mu }}_{\;}^{\text{KM}}$ (0.261×10^−3^) was higher than ${\hat{\mu }}_{\;}^{\text{RL}/2}$ (0.233×10^−3^). The value of ${\hat{\mu }}_{\;}^{\text{KM}}$ was similar to ${\hat{\mu }}_{\;}^{\text{ROS}}$ (0.257×10^−3^) and ${\hat{\mu }}_{\;}^{\text{MLE}}$ (0.266×10^−3^). The value of ${\hat{\sigma }}_{\;}^{\text{KM}}$ (0.338×10^−3^) was similar to ${\hat{\sigma }}_{\;}^{\text{RL}/2}$ (0.353×10^−3^) and ${\hat{\sigma }}_{\;}^{\text{ROS}}$ (0.343×10^−3^). Since the data used herein have more than 50% nondetects, KM estimates may be less relevant.

#### 3.1.4 Regression on order statistics

${\hat{\mu }}_{\;}^{\text{ROS}}$ (0.257×10^−3^) was relatively close to what was obtained using other methods except for DN. ${\hat{\sigma }}_{\;}^{\text{ROS}}$ (0.355×10^−3^) was lower than ${\hat{\sigma }}_{\;}^{\text{MLE}}$ (0.846×10^−3^) and ${\hat{\sigma }}_{\;}^{\text{MCMC}}$ (1.06×10^−3^).

#### 3.1.5 Maximum likelihood estimation

${\hat{\mu }}_{\;}^{\text{MLE}}$ (0.266×10^−3^) was comparable to what was obtained using other methods except for DN. However, ${\hat{\sigma }}_{\;}^{\text{MLE}}$ (0.846×10^−3^) and ${\hat{s}}_{\;}^{\text{MLE}}$ (4.72) were higher compared to the other methods except for MCMC.

#### 3.1.6 Markov chain Monte Carlo

**Fig. 1.**
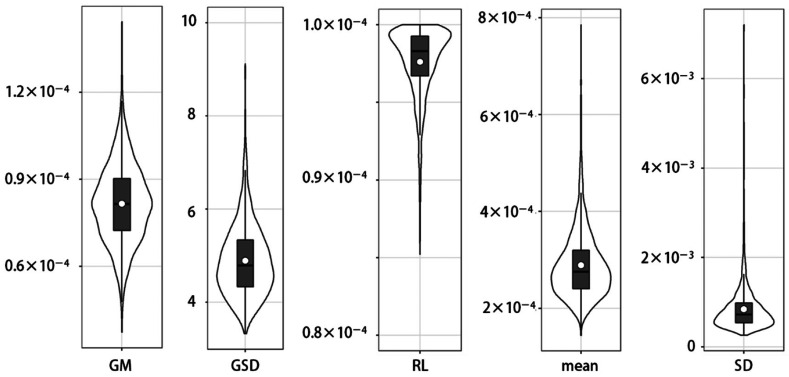
Violin plots for posterior predictive distribution of parameters (geometric mean (GM), geometric standard deviation (GSD), reporting limit (RL), mean, and standard deviation (SD)) about Cr(VI) concentration in mineral water products reported by Kataoka et al (2017) under assumption that original data follow a lognormal distribution. Violins, boxes, horizontal solid lines, and open circles indicate probability density, interquartile range, median, and arithmetic mean, respectively.

The posterior predictive distributions of parameters (GM, GSD, and RL) and generated quantities (means and SD) are shown in Fig. 1. The value of ${\hat{\mu }}_{\;}^{\text{MCMC}}$ (0.289×10^−3^) was slightly higher compared to KM, ROS, and MLE. ${\hat{m}}_{\;}^{\text{MCMC}}$ (0.082×10^−3^) showed the second lowest value among all other methods. ${\hat{\sigma }}_{\;}^{\text{MCMC}}$ (1.06×10^−3^) and ${\hat{s}}_{\;}^{\text{MCMC}}$ (4.94) were higher than corresponding values from all other methods.

The 95% CrI for the mean by MCMC was wider than other methods. There are two possible reasons for this. The first is that, according to the Cox method, the larger GSD resulted in the wider CrI. Another reason is that the estimated parameters themselves have a distribution in MCMC, and the uncertainty was combined, resulting in wider CrI than other methods.

#### 3.1.7 Distribution of Cr(VI) concentrations in mineral water products

**Fig. 2.**
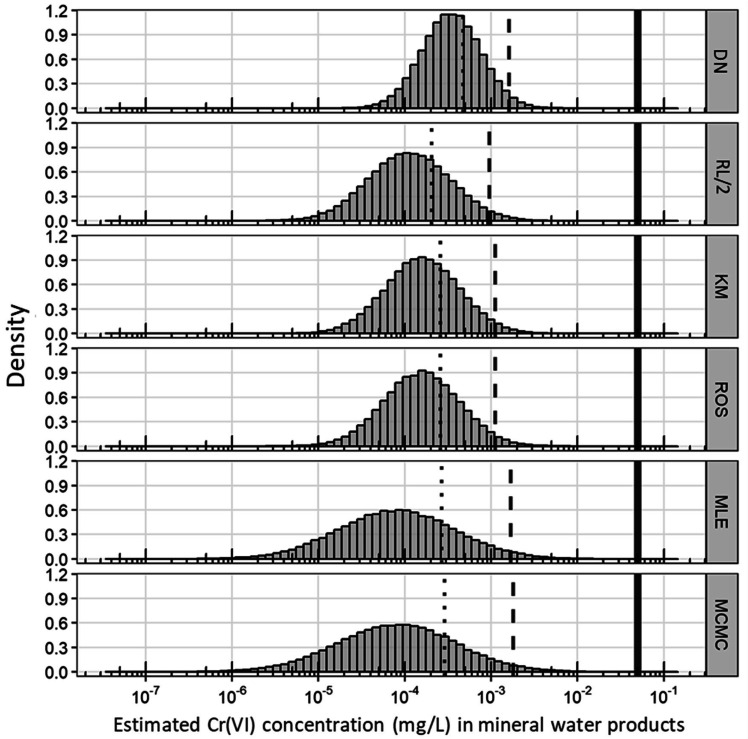
Histograms of generated random numbers for Cr(VI) concentrations in mineral water products following a lognormal distribution with estimated parameters by DN, RL/2, KM, ROS, MLE, and MCMC. Vertical solid, dashed, and dotted lines indicate regulated concentration (0.05 mg/L), 97.5^th^ percentiles, and mean, respectively.

To estimate the distribution of Cr(VI), a total of 10^5^ Cr(VI) random numbers were generated (Fig. 2). Regardless of estimation method, Cr(VI) concentrations in MW products were sufficiently lower than the regulated value of 0.05 mg/L stipulated by the Food Sanitation Act^42^^)^. The mean values were higher in the order of DN (0.472×10^−3^) > MCMC (0.289×10^−3^) > MLE (0.268×10^−3^) > KM (0.260_2_×10^−3^) > ROS (0.259_9_×10^−3^) > RL/2 (0.205×10^−3^). The 97.5^th^ percentile values were higher in the order of MCMC (1.82×10^−3^) > MLE (1.69×10^−3^) > DN (1.61×10^−3^) > ROS (1.12_3_×10^−3^) > KM (1.11_7_×10^−3^) > RL/2 (0.96×10^−3^). Since the actual 97.5^th^ percentile value is 1.38×10^−3^, it can be concluded that the methods explored herein express the original data reasonably well. The estimated mean values showed similar values except for DN, but the 97.5^th^ percentile value was larger when MCMC was used.

### 3.2 Comparison the Different Methods

Since the true values for parameters of probability density distribution are unknown, we conducted the simulation study to determine the most appropriate methods. We compared the performances of each method with using assessment criteria (bias, MSE, CP, RIQR, and RSD). Ideally, we want the methods being studied to have a bias as close to 0 as possible and an MSE as small as possible. In addition to high accuracy, we want to know that a particular method is appropriate in terms of estimating CI for means, and reliable when faced with alternative data sets.

#### 3.2.1 Accuracy

**Fig. 3.**
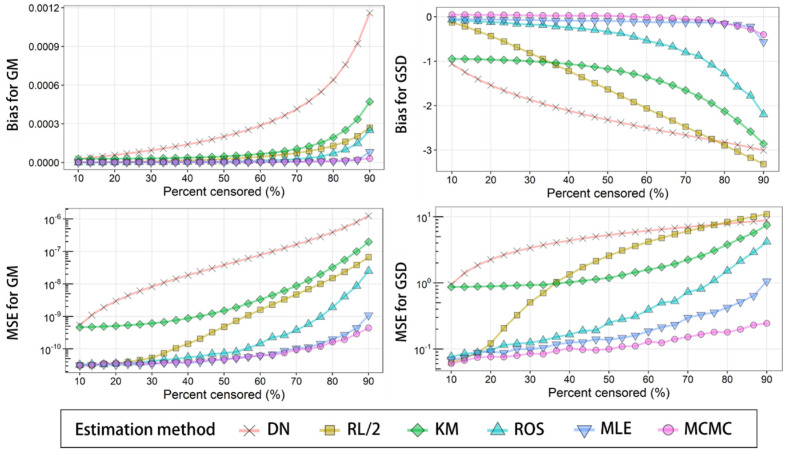
Bias and mean squared error (MSE) for GM and GSD estimated by DN, RL/2, KM, ROS, MLE, and MCMC from 1000 randomly generated left-censored data subsets (*n *= 150) which follow a lognormal distribution with certain parameters (GM = 0.082×10^−3^; GSD = 4.9) over a 10 to 90% censoring range. For MCMC, the expected a posteriori was used for further calculation.

The results of accuracy assessment under censored percentages from 10 to 90% are shown in Fig. 3. The DN method overestimates GM and underestimates GSD. This tendency became more pronounced as the censored percentage increases. We should refrain from calculating summary statistics after discarding nondetects. MCMC tended to overestimate the GSD slightly (<101% overestimated when the censored percentage was between 10 and 50), but other methods tended to underestimate the GSD.

**Fig. 4.**
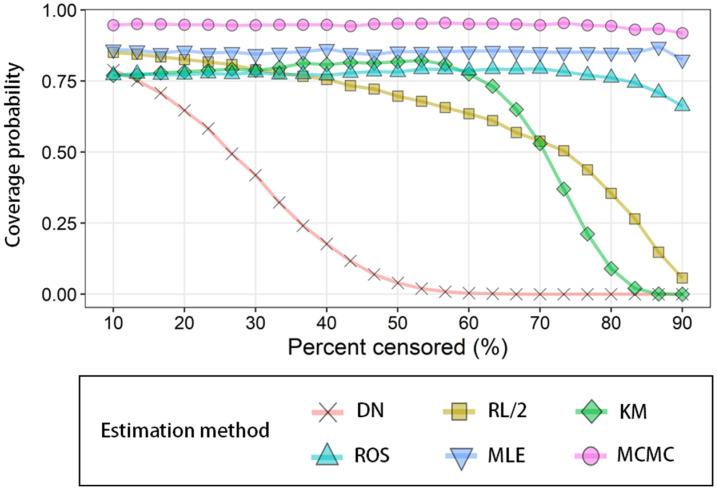
Coverage probability of 95% CI or 95% CrI for mean estimated by DN, RL/2, KM, ROS, MLE, and MCMC from 1000 randomly generated left-censored data subsets (*n *= 150) which follow a lognormal distribution with certain parameters (GM = 0.082×10^−3^; GSD = 4.9) over a 10 to 90% censoring range.

Even when the censored percentage is small, ${\hat{s}}_{\;}^{\text{DN}}$ and ${\hat{s}}_{\;}^{\text{KM}}$ exhibited high MSE values. Where the censored percentage was between 10 and 50, the ROS, MLE, and MCMC methods exhibited similar MSE values for both GM and GSD. However, in terms of the ROS method, MSE increased from around 50% censoring upwards for both GM and GSD.

${\hat{m}}_{\;}^{\text{MLE}}$ and ${\hat{m}}_{\;}^{\text{MCMC}}$ showed similar values in the censoring range of 10 to 90%, and these estimation methods exhibited better accuracy. The MSE of both ${\hat{m}}_{\;}^{\text{MCMC}}$ and ${\hat{s}}_{\;}^{\text{MCMC}}$ were lower compared to other estimation methods when the censored percentage was in the range of 10 to 90%. From these results, we concluded that MCMC showed the best accuracy to estimate distribution parameters from the original data.

#### 3.2.2 Coverage probability

The results of CPs assessment under censored percentages from 10 to 90% are shown in Fig. 4. The CPs decreased sharply from 10% for DN and 60% for the KM method. In terms of the RL/2 method, the CP gradually decreased from 10%, and rapidly decreased around 70%. In ROS and MLE, the CP showed similar values (75 to 85%) depending on the censoring ratio. MCMC showed the most stable CPs, and the CPs were generally close to the target coverage of 0.95.

#### 3.2.3 Reliability

**Fig. 5.**
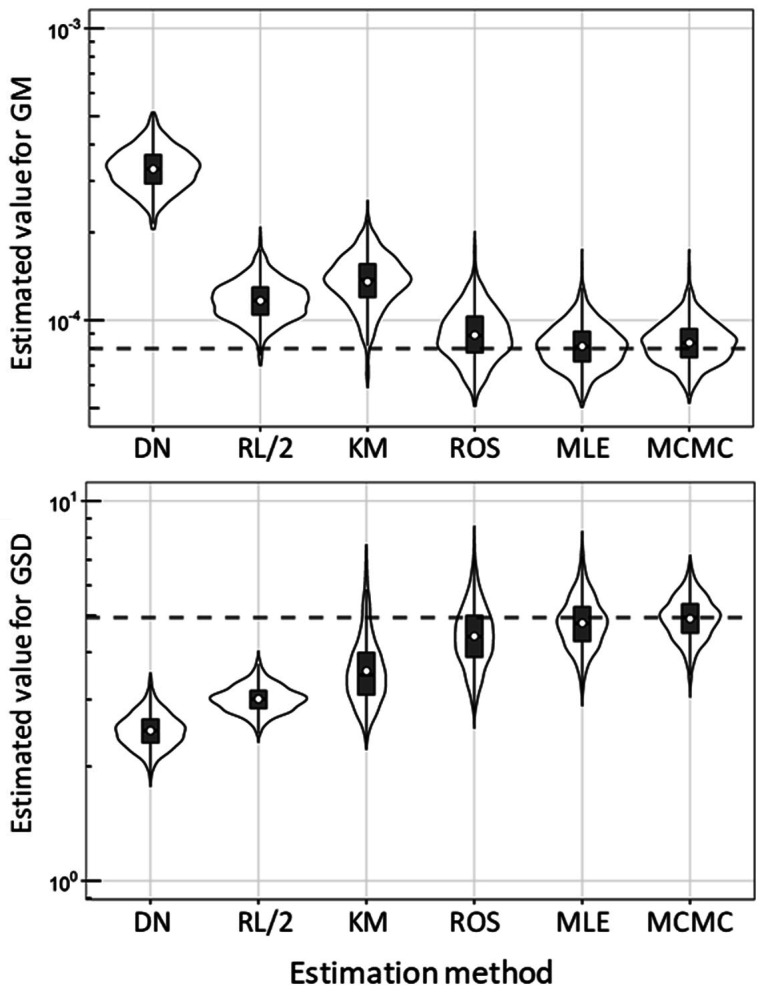
Violin plots of GM and GSD estimated by DN, RL/2, KM, ROS, MLE, and MCMC from 1000 randomly generated left-censored data subsets (*n *= 150) which follow a lognormal distribution with certain parameters (GM = 0.082×10^−3^; GSD = 4.9) at a censoring ratio of 85/150. For MCMC, the expected a posteriori was used for further calculation. Horizontal dashed lines indicate true values. Violins, boxes, horizontal solid lines, and open circles indicate probability density, IQR, median, and arithmetic mean, respectively.

The violin plots for $\hat{m}$ and $\hat{s}$ at the censored ratio of 85/150 are shown in Fig. 5. The true values ​​were distributed within the IQR estimated by ROS, MLE, and MCMC, but not within those by DN, RL, and KM. Among these three estimation methods, MCMC showed the smallest variation in RIQR and RSD for both $\hat{m}$ and $\hat{s}$.

**Fig. 6.**
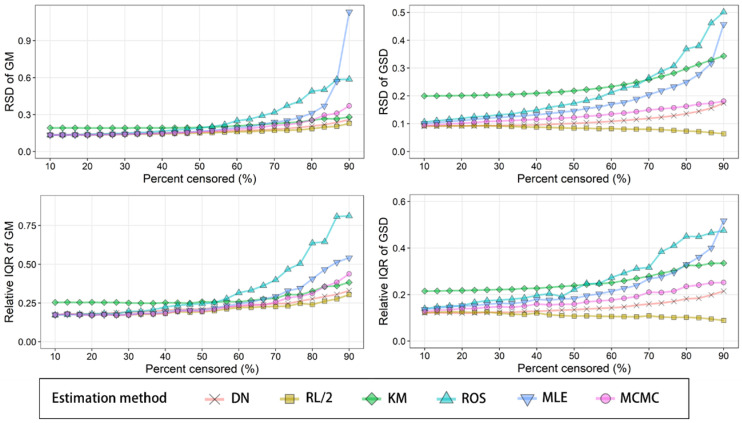
Relative standard deviation (RSD) and relative interquartile range (IQR) of GM and GSD estimated by DN, RL/2, KM, ROS, MLE, and MCMC from 1000 randomly generated left-censored data subsets (*n *= 150) which follow a lognormal distribution with certain parameters (GM = 0.082×10^−3^; GSD = 4.9) over a 10 to 90% censoring range. For MCMC, the expected a posteriori was used for further calculation.

The results of reliability assessment under censored percentages from 10 to 90% are shown in Fig. 6. The variations for both $\hat{m}$ and $\hat{s}$ were generally in the order of RL/2 < DN < MCMC < MLE < ROS. When the censored rate was high, KM exhibited lower RSD and RIQR compared to MLE and ROS. Even when the censored percentage was low, KM exhibited higher variation in both RSD and RIQR compared to other methods.

Since DN, RL/2, and KM showed lower accuracy, when these three methods were excluded, the MCMC estimation exhibited lower variation in both RSD and RIQR compared to ROS and MLE at censored percentages from 10 to 90%. From these results, it was concluded that MCMC is the most reliable estimation method.

Although ${\hat{s}}_{\;}^{\text{MCMC}}$ showed reasonable reliability (the maximum RSD of ${\hat{s}}_{\;}^{\text{MCMC}}$ was 0.18 at 90% censoring), the variation in ${\hat{m}}_{\;}^{\text{MCMC}}$ was as high as RSD=0.37 and RIQR=0.44 at a censoring rate of 90%. A reliable estimation method for left-censored data with a high censoring rate is required.

## 4. Discussions

We have described the MCMC method, as well as the commonly used methods, for summarizing and analyzing left-censored Cr(V) concentration in MW products. We used a simulation study to demonstrate the properties of these methods. MCMC showed the most stable CPs with the highest accuracy, and the CPs were generally close to the target coverage of 0.95. These results are consistent with previous reports^32^^,^^34^^)^. MLE showed the second-best performance next to MCMC. Since the original data is sufficient for MLE requirements (more than 50 observations and censoring ratio of 50–80%) recommended by Helsel^18^^)^, the performance of MLE was superior to those of ROS and KM. Other studies have also reported that MLE has a smaller bias than ROS (or probability plotting method) and KM^25^^,^^27^^)^. Although RL/2 showed the best reliability, RL/2 showed lower accuracy and coverage probability under censored percentage from 20 to 90%. The RL/2 estimation results are considered to have a serious bias. As reported previously, substitution is essentially creating data which does not have an empirical basis. Since our simulation results are consistent with previous reports, the simulation in this study is considered to be valid.

Although the reliability of Bayesian approaches on left-censored data is less understood, our study shows that MCMC performed with the smaller variation in estimates compared to KM, ROS, and MLE. Since MCMC showed better performance on accuracy, coverage probability, and reliability compared to other methods, estimates by MCMC for Cr(VI) concentration in MW products are the most appropriate estimates among the methods used in this study. The most appropriate estimates of mean and those of 97.5^th^ percentile of Cr(VI) concentration, which were estimated with MCMC, were 0.289×10^−3^ and 1.82×10^−3^ mg/L, respectively. These estimates were sufficiently lower than the regulated value of 0.05 mg/L stipulated by the Food Sanitation Act^42^^)^.

Huynh et al^31^^)^ reported that the use of more informative priors generally improved the Bayesian method’s performance, making both the bias and the root MSE lower. The adoption of the appropriate prior distribution is sometimes difficult under the conditions when they are needed most, i.e. small sample sizes or high censored percentage. Since MCMC showed better performance on accuracy, coverage probability, and reliability, we have successfully adopted informative prior distribution to the original data. We consider that this is the main reason why the MCMC showed better performance compared to other methods. It is well known that MCMC can accommodate outliers by describing data via heavy-tailed distributions to the extent implied by the data^43^^)^. Since lognormal distributions with GSD greater than 4 are considered to have heavy tails, the performance of MCMC was superior to that of the other methods. In this study, we used the EAPs as point estimator in MCMC. Other point estimators (maximum a posteriori and median of posterior predictive distribution) for Bayesian estimation might show different performances. These are other reasons why the MCMC is better than other methods.

To perform safe assessment of exposure to chemicals, substituting RL for nondetects may be a conservative and preferable method. However, when the dissociation between reality and assumption is large, such a conservative method may generate overestimates that are meaningless for health risk, leading to false concern and excessive regulation. Rather than imposing strict rules onto society and the food community, it is important to introduce new, scientifically sound methods and resolve the lack of transparency arising from inadequate evaluation. The use of prior distributions in Bayesian estimation can be considered as bridging a prior knowledge to MLE. Although it is necessary to carefully check whether the prior distribution has been adopted appropriately, utilizing knowledge of society and food community, as well as researchers, is able to help to reduce the gap between science-technology and society. Bayesian estimation provides the whole (posterior predictive) distribution for each parameter estimated, instead of one single-value estimate in MLE, thus it can provide estimates on the safe side with considering uncertainty. We expect that Bayesian estimation with incorporating consumer and regulatory science reduce this gap in food safety fields.

Since the original data are univariate, we have not extended the regression model in this study. However, it may be possible to estimate individual values ​​of nondetects using data covering signal intensity, production location, and other information. If sufficient information is available on the manufacturing process, storage method, factory, manufacturer, and so on, a hierarchical Bayesian model that considers these factors as random effects and incorporates them into the model could be considered (see **Supporting Information S8. **as example of comparison between Japanese and imported MW products). By virtue of such a complex analysis, it may be possible to evaluate manufacturing process risks such as contamination or changes in chemical species.

This study demonstrates that using the MCMC method provide the most appropriate estimation result for Cr(VI) concentration in MW products. On the other hands, as mentioned in many articles^18^^,^^27^^,^^44^^)^, our simulation result indicate that the use of RL/2 (and DN method) should be avoided. Evaluation, prediction, and judgment based on scientific evidences are the basis of regulatory science and are important for risk assessment in food safety administration. MCMC, as well as other methods based on statistical validity such as MLE, is important in the field of food safety as an estimation method instead of conventional substitution method. On the other hand, it should be discussed carefully whether the MCMC estimation results are appropriate for other data. Evaluation of the validity for different probability density distributions, sample sizes, and parameters is necessary.

## 5. Conclusions

We demonstrate that parameter estimation from left-censored data with MCMC show better performance on accuracy, coverage probability, and reliability compared to other methods (DN, RL/2, KM, MLE, and ROS) for Cr(VI) concentration in MW products. The most appropriate estimate of mean concentration, which was estimated with MCMC, was 0.289×10^−3^ mg/L and this estimate was sufficiently lower than the regulated value of 0.05 mg/L stipulated by the Food Sanitation Act.

In the field of food safety, although chemical substances in food are rarely detected, there are many chemical substances that have to be periodically investigated for protecting public health. This study suggests that MCMC could be a powerful estimation method in exposure assessment contexts in food safety science. However, there is ample scope of future research for the standardization to explore this further for the model with various sample size, alternative parameters, and different distributions, as well as multiple RLs due to the performance difference of analytical instruments.

## Supporting Information


