# Supplementary material for: Attempt of Bayesian Estimation from Left-censored Data Using the Markov Chain Monte Carlo Method: Exploring Cr(VI) Concentrations in Mineral Water Products
Source: Food Saf (Tokyo). 2020 Dec 25;8(4):67–89. doi: 10.14252/foodsafetyfscj.D-20-00007 (PMC7765759; doi:10.14252/foodsafetyfscj.D-20-00007)
Supplement: Supplementary file 1 [file foodsafetyfscj-8-67-s001.pdf]

doi: 10.14252/foodsafetyfscj.D-20-00007

Supporting Information

S1. Description of the Stan Program

The Stan code assuming a lognormal distribution is shown in **Fig. S1**. This code contains 5 blocks (data, transformed data, parameter, model, and generated quantities). In the data block (**Fig. S1**, lines 1-6), we specified 3 data dimensions (sample number of observed (N_{obs}) and censored (N_{cen}) data, and value of observed data (Y_{obs})). Moreover, we specified repetition number to generate random numbers (N_{new}) for 2-dimensional Monte Carlo simulations. In the transformed data block (**Fig. S1**, lines 8–14), we calculated the minimum and maximum value of Y_{obs}. In the parameter block (**Fig. S1**, lines 16–21), we declared 3 parameters (GM, GSD, and RL) and 85 non-detected values (Y_{cen}) to estimate.

In the model block (**Fig. S1**, lines 23–31), Stan can estimate declared parameter(s). In the Bayesian framework, all parameters follow a probability distribution, so a prior distribution must be specified. In lines 24–27 of **Fig. S1**, the informative prior distributions for all parameters are specified. Since GM is equivalent to median of lognormal distribution, we used the normal distribution with mean of half maximum value of Y (Y_{max}) and standard deviation of $Y_{max}/4$. The natural total Cr content of dissolved waters is approximately 0.02–0.3 μg/L[1). If the Cr concentration follows a logarithmic distribution and this range covers 80% of the concentration distribution, the GSD is estimated to be 2.9. Thus, we used the lognormal distribution with GM of 3 and GSD of 2 for prior distribution of GSD. It is expected that RL is close to minimum of observed data. We used the normal distribution with mean of minimum value of Y_{obs} ($Y_{obs,\ min}$) and standard deviation of $Y_{obs,\ min}/5$. In lines 29 and 30 of **Fig. S1**, both Y_{obs} and Y_{cen} are specified as stochastically generated from a lognormal distribution with certain parameters, and Stan seeks to find the optimum parameter values from the data.

In the generated quantities block (**Fig. S1**, lines 33–45), we calculated the posterior predictive distribution of log(arithmic) pointwise density for both observed and censored data. For the likelihoods of censored data, we used the cumulative distribution function (CDF). Moreover, in this block, we generated random numbers according to the declared distribution having estimated parameters.

In addition to lognormal distribution, we performed Bayesian estimation under assumption that the original data follow a gamma distribution with the stan code in **Fig. S2**.

S2. Convergence of MCMC

After the MCMC iteration finished, the three parameters (GM, GSD, and RL) declared in the parameter block and the sum of the log(arithmic) posterior predictive probabilities (lp__) had converged (**Figs. S3a and b**). Besides these four values, in all calculation results such as log posterior predictive probabilities, $\hat{R}$, which is the ratio of inter-chain variance to intra-chain variance, satisfied 1.1 or less, which is a general criterion of convergence. In addition, the relative effective sample number and the relative Monte Carlo standard deviation also satisfied general criteria (0.1 or more and 0.1 or less, respectively) (**Fig. S3c**).

In addition, the estimated values about these parameters were similar even assuming different prior distributions (**Table S1**). From these results, the dissociation of the estimated values among the chains and the influence of autocorrelation are small, and each chain started from different initial values and finally arrived at a similar value. We conclude that all the calculated values converged. Even when assuming a gamma distribution, parameter convergence was confirmed under identical conditions.

S3. Calculation of Widely Applicable Information Criterion (WAIC)

The widely applicable information criterion (WAIC) derived by Watanabe[2) as an information criterion for Bayesian inference, is defined as follows:

$$\mathrm{WAIC} = L_{\mathrm{WAIC}} + p_{\mathrm{WAIC}}, \qquad \text{Equation S1}$$

where L_{WAIC} denotes average of the pointwise predictive density, which shows the Bayes training loss, and p_{WAIC} denotes fluctuation of the posterior distribution, which indicates the estimated effective number of parameters.

L_{WAIC} and p_{WAIC} are defined as follows:

$$L_{\mathrm{WAIC}} = -\frac{1}{n}\sum_{i=1}^{n} \log E\left(L_i\right), \qquad \text{Equation S2}$$

$$p_{\mathrm{WAIC}} = \frac{1}{n}\sum_{i=1}^{n} V\left(\log L_i\right), \qquad \text{Equation S3}$$

Where n represents the sample size, L_i represents the point-

```
1.  data {
2.      int N_obs; // sample number of detected data
3.      int N_cen; // sample number of nondetects
4.      real<lower=0> Y_obs[N_obs]; // Values of detected data
5.      int N_new;
6.  }
7.
8.  transformed data {
9.      real<lower=0> Y_obs_min; // minimum of observed value
10.     real<lower=0> Y_obs_max; // maximum of observed value
11.
12.     Y_obs_min = min(Y_obs);
13.     Y_obs_max = max(Y_obs);
14. }
15.
16. parameters {
17.     real<lower=0> GM; // location parameter, geometric mean
18.     real<lower=1> GSD; // shape parameter, geometric standard deviation
19.     real<lower=0, upper=Y_obs_min> RL; // Reporting limit
20.     real<lower=0, upper=RL> Y_cen[N_cen]; // Values of nondetects
21. }
22.
23. model {
24.     GSD ~ lognormal( log(3), log(2) );
25.     GM ~ normal(Y_obs_max/2, Y_obs_max/4);
26.     RL ~ normal(Y_obs_min, Y_obs_min/5);
27.     Y_cen ~ uniform(0, Y_obs_min);
28.
29.     Y_obs ~ lognormal( log(GM), log(GSD) );
30.     Y_cen ~ lognormal( log(GM), log(GSD) );
31. }
32.
33. generated quantities {
34.     real log_lik_obs[N_obs];
35.     real log_lik_cen[N_cen];
36.     vector[N_new] Y_new;
37.     real mean_est;
38.     real sd_est;
39.
40.     for (i in 1:N_obs) log_lik_obs[i] = lognormal_lpdf(Y_obs[i] | log(GM), log(GSD));
41.     for (i in 1:N_cen) log_lik_cen[i] = lognormal_lcdf(RL | log(GM), log(GSD));
42.     for (i in 1:N_new) Y_new[i] = lognormal_rng( log(GM), log(GSD) );
43.     mean_est = exp( log(GM) + 0.5*( log(GSD) )^2 );
44.     sd_est = sqrt( exp( 2 *log(GM) + (log(GSD))^2 ) * (exp( (log(GSD))^2 )-1) );
45. }
46.
```

Fig. S1. Stan code for estimating parameters from left-censored data assuming a lognormal distribution

wise predictive density for each data point i obtained from the posterior predictive distribution, $E(X)$ indicates the mean of variable X, and $V(X)$ indicates the variance of variable X. To calculate complete likelihood according to the likelihood function (Equation 1), we used log(arithmic) pointwise density (**Fig. S1**, line 40) for log L_i of observed data and log(arithmic) lower-tail probability (**Fig. S1**, line 41) for log L_i of censored data.

Unlike other criteria, WAIC has a theoretical foundation as follows[2]:

$$E(G) \equiv E(\mathrm{WAIC}) + O\left(\frac{1}{n}\right), \qquad \text{Equation S4}$$

doi: 10.14252/foodsafetyfscj.D-20-00007

```
data {
    int N_obs; // sample number of observed data
    int N_cen; // sample number of censored data
    real<lower=0> Y_obs[N_obs]; // Values of detected data
    int N_new;
}

transformed data {
    real<lower=0> Y_obs_min; // minimum of observed value
    real<lower=0> Y_obs_max; // maximum of observed value

    Y_obs_min = min(Y_obs);
    Y_obs_max = max(Y_obs);
}

parameters {
    real<lower=0> shape; // shape parameter of gamma distribution
    real<lower=0> rate; // rate parameter of gamma distribution
    real<lower=0, upper=Y_obs_min> RL; // Reporting limit
    real<lower=0, upper=RL> Y_cen[N_cen]; // Values of nondetects
}

model {
    shape ~ double_exponential(1, 5);
    target += normal_lpdf( shape/rate | Y_obs_max/2, Y_obs_max/4 );
    RL ~ normal(Y_obs_min, Y_obs_min/5);
    Y_cen ~ uniform(0, Y_obs_max);

    Y_obs ~ gamma(shape, rate);
    Y_cen ~ gamma(shape, rate);
}

generated quantities {
    real log_lik_obs[N_obs];
    real log_lik_cen[N_cen];
    vector[N_new] Y_new;
    real mean_est;
    real sd_est;

    for (i in 1:N_obs) log_lik_obs[i] = gamma_lpdf( Y_obs[i] | shape, rate);
    for (i in 1:N_cen) log_lik_cen[i] = gamma_lcdf( RL | shape, rate);
    for (i in 1:N_new) Y_new[i] = gamma_rng(shape, rate);
    mean_est = shape/rate;
    sd_est = sqrt(shape)/rate;
}

```

Fig. S2. Stan code for estimating parameters from left-censored data assuming a gamma distribution

Where G represents Bayes generalization loss and $O(g(x))$ represents mathematical notation that describes the limiting behavior of a function ($f(x)$) when the argument tends toward a particular value or infinity. The expectation value of the Bayes generalization loss is asymptotically equal to WAIC. And Watanabe[2] also showed the cross-validation loss is asymptotically equivalent to the WAIC, even in singular statistical model. WAIC can be used for any true distribution, probability model, and prior distribution. The WAIC is a generalized version of Akaike's information criterion (AIC) and can be calculated from posterior predictive distributions by MCMC.

S4. Probability Distribution Assumption

(a)

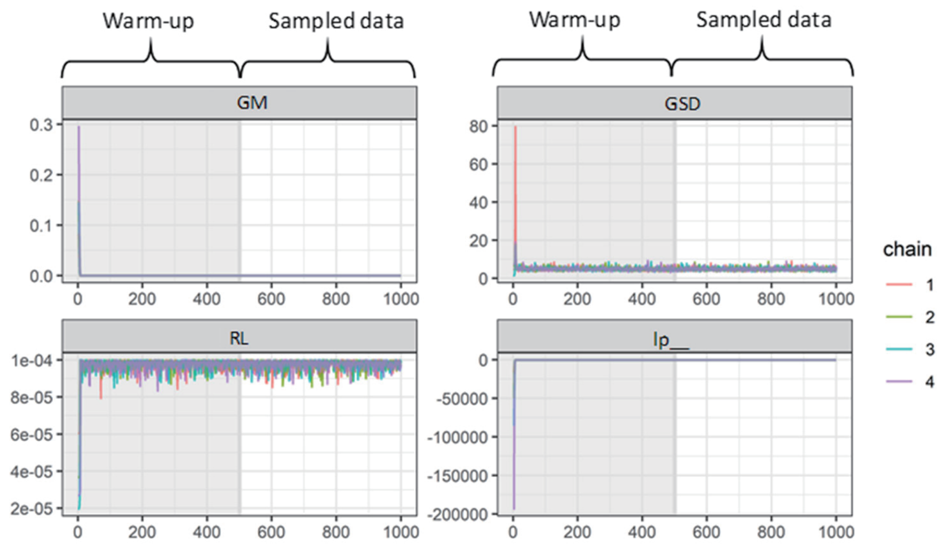

(b) Convergence indices for MCMC parameters (iterations=2000, warm-up=1000, chain=4, thinning=2)

Parameter	Mean	SD	$\hat{R}$	n_{eff}/N	mcse/sd
GM	0.082×10^{-3}	0.014×10^{-3}	1.001	0781	0.025
GSD	4.90	0.80	0.999	0.739	0.026
RL	0.098×10^{-3}	0.002×10^{-3}	0.9996	0.874	0.024
lp__	−225	8.4	1.001	0.475	0.032

(c)

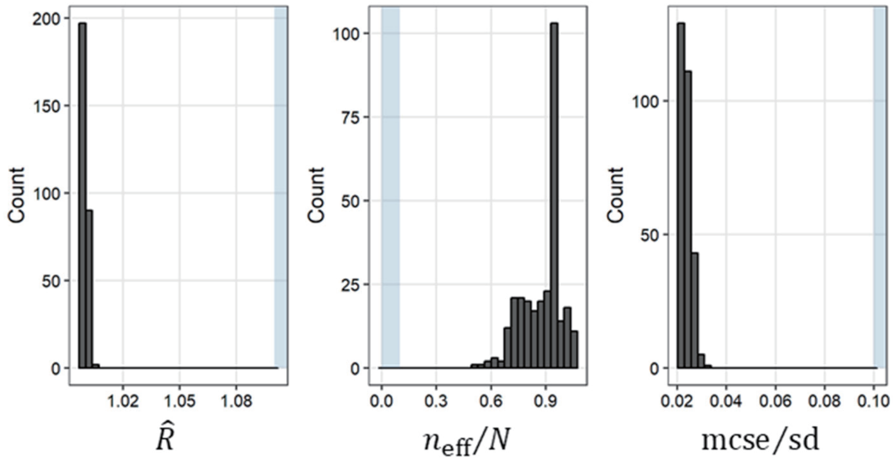

Fig. S3. Confirmation of MCMC convergence. (a) Trace plot for declared parameters (GM, GSD, and RL) in stan code (**Fig. 1**) and the sum of log posterior predictive probabilities (lp__); (b) Summary of estimation and convergence indices for parameters and lp__; (c) Histograms of three convergence indices ($\hat{R}$, n_{eff}/N, and mcse/sd) for all calculated values, where $\hat{R}$ is the ratio of intra-chain variation to inter-chain variance, n_{eff}/N is the ratio of effective sample number to MCMC sample, and mcse/sd is the ratio of Monte Carlo standard error to standard deviation. If calculated data are not distributed in the light blue area, it can be concluded that MCMC calculations have converged.

doi: 10.14252/foodsafetyfscj.D-20-00007

Table S1. MCMC results assuming a lognormal distribution using various prior distributions. Mean ± standard deviation and 95% credible interval are shown.

Prior distribution	GM (× 10^{-3})	GSD	WAIC
GM ~ normal(Y_max/2, Y_max/4)[a*]<br>GSD ~ lognormal(log(3), log(2))[b*]	0.082±0.014<br>0.055–0.111	4.90±0.80<br>3.65–6.71	−2.28
GM ~ cauchy(0, 5)[c]<br>GSD ~ lognormal(log(3), log(2))	0.081±0.013<br>0.057–0.108	4.94±0.80<br>3.76–6.90	−2.28
GM ~ uniform(0, 10^5)[d]<br>GSD ~ uniform(0, 10^5)	0.078±0.014<br>0.052–0.106	5.24±0.98<br>3.88–7.61	−2.28
GM ~ normal(0, 10^5)<br>GSD ~ normal(0, 10^5)	0.078±0.014<br>0.054–0.109	5.20±0.93<br>3.83–7.36	−2.28
GM ~ cauchy(0, 5)<br>GSD ~ cauchy(0, 5)	0.079±0.014<br>0.053–0.108	5.08±0.87<br>3.81–7.04	−2.28

a: "$X \sim$ normal(μ, σ)" means that variable X follows a normal distribution with location parameter μ and shape parameter σ.
b: "$X \sim$ lognormal(μ, σ)" means that variable X follows a lognormal distribution with location parameter μ and shape parameter σ.
c: "$X \sim$ cauchy(μ, σ)" means that variable X follows a Cauchy distribution with location parameter μ and shape parameter σ.
d: "$X \sim$ uniform(a, b)" means that variable X follows a uniform distribution with range from a to b.
* The prior distributions used in **Fig. 1**.

Some EPA guidance documents strongly recommend against using a lognormal model for environmental data and instead suggest a gamma distribution[3]. We examined whether a lognormal or a gamma distribution is more appropriate for the original data. The WAIC assuming a lognormal and a gamma distribution resulted in similar values for WAIC (**Table S2**).

The WAIC (−2.28) of original data assuming a lognormal distribution was in good agreement with WAICs (−2.33±0.09) from 1000 randomly generated left-censored data subsets (n = 150) which follow a lognormal distribution with certain parameters (GM = 0.082×10^{-3}; GSD = 4.9) at a censoring ratio of 85/150 (**Fig. S4**). This result indicate that the data fit a lognormal distribution reasonably well.

Although WAIC assuming a lognormal distribution was slightly higher than that assuming a gamma distribution (−2.29), to compare as many estimation methods as possible, we proceeded with further analysis under the assumption that the original data follow a lognormal distribution.

S5. Cox's Method for 95%CIs for Means Which Follow a lognormal Distribution

The 95% CIs for means estimated by DN and RL/2 were calculated by Cox's method[4] as follows:

$$\left[\hat{\mu}/\exp\left\{1.96\sqrt{\frac{\hat{s}^2}{n}+\frac{\hat{s}^4}{2(n-1)}}\right\},\ \hat{\mu}\times\exp\left\{1.96\sqrt{\frac{\hat{s}^2}{n}+\frac{\hat{s}^4}{2(n-1)}}\right\}\right],$$

Equation S5

where $\hat{\mu}$ indicates the estimated mean, $\hat{s}$ denotes the log-transformed GSD estimate, and n is total observations.

S6. Estimation Using Left-censored Data

S6.1 The Kaplan-Meier Algorithm

The Kaplan-Meier (KM) method is a nonparametric technique for dealing with censored data. It is widely used in survival and lifetime data analysis to estimate survival functions, which are then used to estimate different summary statistics. The KM method can be summarized via the following steps:[5]

1) This method requires the use of right-censored data. Therefore, right-censored data (Flip_i) are constructed by subtracting all observations (x_i) from M.

$$\text{Flip}_i = M - x_i,$$ Equation S6

Where M equals a flipping constant larger than the maximum of x_i.

2) The survival function probability ($S(t)$) is the product of the j = 1 to k incremental survival probabilities to the point, proceeding from high to low concentration for the k uncensored observations.

$$S(t_j) = \prod_{j=1}^{k}\frac{b_j - d_j}{b_j},$$ Equation S7

Where t_j is the flipped uncensored observations; b_j is the

Table S2. MCMC results assuming both lognormal and gamma distributions for estimating censored values. Widely applicable information criterion, mean ± standard deviation, maximum a posteriori (MAP), and 95% credible interval (CrI) of posterior predictive distribution for target parameters

	Lognormal	Gamma
WAIC	−2.28	−2.29
Parameter	RL (× 10^{-3})<br>0.098±0.002<br>MAP: 0.099_3, 95% CrI: 0.091–0.099_9	RL (× 10^{-3})<br>0.096±0.004<br>MAP: 0.099_0, 95% CrI: 0.087–0.099_9
	GM (× 10^{-3})<br>0.082±0.014<br>MAP: 0.080, 95% CrI: 0.055–0.111	Shape<br>0.353±0.059<br>MAP: 0.354, 95% CrI: 0.254–0.487
	GSD<br>4.90±0.80<br>MAP: 4.60, 95% CrI: 3.65–6.71	Rate<br>1625±347<br>MAP: 1589, 95% CrI: 1019–2378
	Mean (× 10^{-3})<br>0.290±0.71<br>MAP: 0.249, 95% CrI: 0.197–0.459	Mean (× 10^{-3})<br>0.221±0.31<br>MAP: 0.212, 95% CrI: 0.168–0.288
	SD (× 10^{-3})<br>1.09±0.70<br>MAP: 0.715, 95% CrI: 0.453–2.88	SD (× 10^{-3})<br>0.375±0.60<br>MAP: 0.353, 95% CrI: 0.275–5.16

number of observations, both detected and censored, at and higher than each t_j; and d_j is the number of uncensored observations at that value.

3) The KM estimate of the mean is calculated by integrating the area under the KM survival curve. Since this is the average value for flipped right-censored data, the average value ($\hat{\mu}^{\mathrm{KM}}$) for the original left-censored data is calculated as follows:

$$\hat{\mu}^{\mathrm{KM}} = M - \int_{0}^{t_{\max}} \hat{S}(t)\,dt \approx M - \sum_{j=1}^{k} \left\{ S(t_{j-1}) \times (t_j - t_{j-1}) \right\}. \quad \text{Equation S8}$$

4) Following Lee and Wang[6)], the variance of the mean is computed using the formula inside the square root sign of the following equation for data with m censored values under the Kaplan-Meier curve:

$$\text{Standard error}^{\mathrm{KM}} = \sqrt{\left(\frac{m}{m-1}\right) \sum_{r=1}^{m} \frac{A_r^{\,2}}{(n-r)(n-r+1)}}, \quad \text{Equation S9}$$

Where n is total observations and A_r is the cumulative area. Finally, the standard deviation ($\hat{\sigma}^{\mathrm{KM}}$) is calculated as follows:

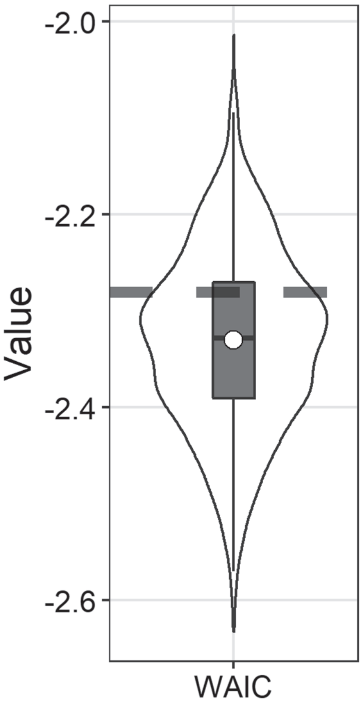

Fig. S4. Violin plot of WAICs from 1000 randomly generated left-censored data subsets (n = 150) which follow a lognormal distribution with certain parameters (GM = 0.082×10^{-3}; GSD = 4.9) at a censoring ratio of 85/150. Horizontal dashed lines indicate WAIC calculated from original data. Violins, boxes, horizontal solid lines, and open circles indicate probability density, interquartile range, median, and arithmetic mean for simulation data, respectively.

doi: 10.14252/foodsafetyfscj.D-20-00007

$$\hat{\sigma}^{KM} = \sqrt{n} \times \text{Standard error}^{KM}. \qquad \text{Equation S10}$$

In the KM method, the location parameter of the geometric mean (GM) and the shape parameter of the geometric standard deviation (GSD) for a lognormal distribution were calculated using $\hat{\mu}^{KM}$ and $\hat{\sigma}^{KM}$ with the following equations.

If x is a random variable following a lognormal distribution with certain parameters (GM and GSD), and GM and GSD are expressed as e^m and e^s, respectively, and the mean $E(x)$ and variance $V(x)$ are expressed as follows:

$$E(x) = \exp\left[m + \frac{s^2}{2}\right], \qquad \text{Equation S11}$$

$$V(x) = \exp\left[2m + s^2\right]\left(\exp\left[s^2\right] - 1\right). \qquad \text{Equation S12}$$

The GM and GSD parameters estimated by the KM method assuming a lognormal distribution can be transformed as follows:

$$\hat{m}^{KM} = \log \frac{\hat{\mu}^{KM}}{\sqrt{\frac{\left(\hat{\sigma}^{KM}\right)^2}{\left(\hat{\mu}^{KM}\right)^2} + 1}}, \qquad \text{Equation S13}$$

$$\hat{s}^{KM} = \sqrt{\log\left(\frac{\left(\hat{\sigma}^{KM}\right)^2}{\left(\hat{\mu}^{KM}\right)^2} + 1\right)}. \qquad \text{Equation S14}$$

S6.2 The Robust Regression on Order Statistics Algorithm

There are two versions of ROS, one is a fully parametric and the other is a semi-parametric robust implementation ROS. These are sometimes confused in the literature[5]. In this study, we used robust ROS (rROS) developed by Helsel and Cohn[7]. Robust ROS is also known as "Imputation Using Quantile-Quantile Regression". This method involves using quantile-quantile regression on the log-transformed observations to fit a regression line. Using this method, a more limited assumption of normal or lognormal distribution is used. The algorithm for the ROS method can be summarized according to the following steps:

1) In general, the probability of exceeding the jth detection limit (${}^{E}P_j$) is

$${}^{E}P_j = {}^{E}P_{j+1} + \frac{A_j}{A_j + B_j}\left[1 - {}^{E}P_{j+1}\right], \qquad \text{Equation S15}$$

where Aj is the number of observations in the range of jth and $(j + 1)^{th}$ reporting limits, and Bj is the number of observations, censored and uncensored, below the jth reporting limit.

When j is the highest reporting limit, ${}^{E}P_{j+1} = 0$ and $A_j+B_j=n$. The number of censored observations below the jth reporting limit is defined as C_j:

$$C_j = B_j - B_{j-1} - A_{j-1}. \qquad \text{Equation S16}$$

2) Plotting positions for uncensored observation ${}^{D}p_j$ can be calculated by

$${}^{D}p_j = \left(1 - {}^{E}P_j\right) + \left(\frac{i}{A_j + 1}\right)\left[{}^{E}P_j - {}^{E}P_{j+1}\right] \text{ for } i = 1 \text{ to } A_j, \qquad \text{Equation S17}$$

and for censored observations ${}^{C}p_j$ are generally given by

$${}^{C}p_j = \left(\frac{i}{C_j + 1}\right)\left[1 - {}^{E}P_j\right] \text{ for } i = 1 \text{ to } C_j. \qquad \text{Equation S18}$$

3) The normal quantiles of the plotting positions are known as the order statistics of the ROS method. Assuming a lognormal distribution, a linear regression of the uncensored observations against the normal quantiles of the uncensored plotting positions is formed as

$$\log\left(x_{obs}\right) = \beta + \alpha \times {}^{D}p_j. \qquad \text{Equation S19}$$

Then the regression equation for predicting the unobserved data can be obtained as

$$\log\left(\hat{x}_{cens}\right) = \beta + \alpha \times {}^{C}p_j. \qquad \text{Equation S20}$$

4) Using the log-transformed detected data ($\log\left(x_{obs}\right)$) and the predicted log-value ($\log\left(\hat{x}_{cens}\right)$), calculate the mean and standard deviation for the log-transformed x, and finally estimates of the geometric mean ($\hat{m}^{ROS}$) and geometric standard deviation ($\hat{s}^{ROS}$) are obtained. After retransforming the log-scale imputed values, compute the usual method of moments estimates of $\hat{\mu}^{ROS}$ and $\hat{\sigma}^{ROS}$.

Gilliom and Helsel[8] reported that the robust ROS method perform better on high-skew distributions than did MLE.

Helsel and Cohn[7] reported that the robust ROS generally produced better estimates for the mean and standard devia-

tion whenever data did not follow the distribution assumed by maximum likelihood.

S6.3 The Maximum Likelihood Algorithm

Maximum likelihood estimation solves a likelihood equation to estimate the parameter(s) using both detected observations and the proportion of data falling below RL[9]. The observed data (x) enter the likelihood function through the probability density function $f(x|\theta)$ and the censored observations can be accounted for by the cumulative distribution function $F(\mathrm{RL}|\theta) = P(x \leq \mathrm{RL}|\theta)$ as follows:

$$L(\theta|x_1,x_2,\cdots,x_n)=\prod_{x\in D} f(x|\theta)\cdot\prod_{x\in C} F(\mathrm{RL}|\theta), \quad \text{Equation S21}$$

where D is the set of all observed values; and C is the set of all left-censored values. MLE finds the parameter values (θ) that maximize the likelihood function against the observations. In the case of lognormal distribution, $f(x|m_{\log},s_{\log})$ and $F(\mathrm{RL}|m_{\log},s_{\log})$ are defined as follows:

$$f(x|m_{\log},s_{\log})=\frac{1}{\sqrt{2\pi}s_{\log}x}\exp\left\{-\frac{(\log x-m_{\log})^2}{2s_{\log}^2}\right\},$$

Equation S22

$$F(\mathrm{RL}|m_{\log},s_{\log})=\frac{1}{2}\mathrm{erfc}\left(-\frac{\log x-m_{\log}}{\sqrt{2}s_{\log}}\right), \quad \text{Equation S23}$$

where $m_{\log}$ is log-transformed geometric mean, $s_{\log}$ is log-transformed geometric standard deviation, and erfc(x) indicates complementary error function.

In the EnvStat package, a transformation is adopted as follows[10]. Let x be a vector of n observations from a lognormal distribution with location parameter μ_x (mean) and scale parameter σ_x (standard deviation). Set $y = \log(x)$. Then y is a vector of observations from a normal distribution with location parameters $\mu_y = m$ ($\mathrm{GM}_x = e^m$) and scale parameter $\sigma_y = s$ ($\mathrm{GSD}_x = e^s$). The maximum likelihood estimators of $\hat{\mu}_x^{\mathrm{MLE}}$ and $\hat{\sigma}_x^{\mathrm{MLE}}$ are given by

$$\hat{\mu}_x^{\mathrm{MLE}}=\exp\left[\hat{m}^{\mathrm{MLE}}+\frac{\left(\hat{s}^{\mathrm{MLE}}\right)^2}{2}\right], \quad \text{Equation S24}$$

$$\hat{\sigma}_x^{\mathrm{MLE}}=\hat{\mu}_x^{\mathrm{MLE}}\sqrt{\left(\hat{\sigma}^{\mathrm{MLE}}\right)^2-1}, \quad \text{Equation S25}$$

where $\hat{\mu}^{\mathrm{MLE}}$ and $\hat{\sigma}^{\mathrm{MLE}}$ denote the maximum likelihood estimators of μ and σ, respectively.

S7. Confidence Interval and Credible Interval

A credible interval (CrI) is an important concept in Bayesian statistics to describe and summarize the uncertainty. In this regards, CrI is quite similar to the frequentist "confidence Intervals (CI)". However, while their goal is similar, their statistical meaning is different.

- 95% CI: with a large number of repeated samples, 95% CI represents 95% frequency (i.e. 95% proportion) of possible confidence intervals that contain the true estimate of the unknown parameter.
- 95% CrI: given the observed data, there is a 95% probability that the true estimate of unknow parameter would lie within the 95% CrI.

Thus, it is inappropriate to compare CI and CrI directly. In this study, to compare uncertainty of mean estimates, we calculated the coverage probability of 95% CIs for mean (CP), which indicates the fraction of computed CIs and CrIs that include the desired but unobservable parameter value.

S8. Comparison between Japanese and Imported MW Products

To compare Cr(VI) concentration in Japanese MW products and imported ones, we used another Stan code (**Fig. S5**). **Fig. S6A** presents histograms of the Cr(VI) concentrations of Japanese and imported MW products based on empirical data. **Table S3** shows the estimated parameters. Both GM and GSD parameters of Japanese and imported MW products (**Table S3**) showed good agreement within $\pm 1\sigma$ with those in **Table 1**.

Fig. S6B shows the distribution of expected values (**Fig. S5**, lines 47 and 48). Although the distribution of mean Cr(VI) concentrations in imported products was wider, the probability that the expected value of Cr(VI) concentrations in imported MW products is higher than in Japanese products was 0.632. Kataoka et al[11] investigated imported MW products from 14 countries and, based on that, the present results may reflect differences in concentration depending on the country of origin.

Finally, 10^5 random numbers for Cr(VI) concentrations in imported MW and Japanese products were generated, and

doi: 10.14252/foodsafetyfscj.D-20-00007

```
data {
    int N_obs_F; // Obseved sample size of foreign domestic product
    int N_obs_J; // Obseved sample size of Japanese product
    int N_cen_F; // Sample size of nondetects of foreign domestic product
    int N_cen_J; // Sample size of nondetects of Japanese product
    real<lower=0> Y_min; // Minimum value of observed data
    vector<lower=Y_min>[N_obs_F] Y_obs_F; // Observed data of foreign domestic product
    vector<lower=Y_min>[N_obs_J] Y_obs_J; // Observed data of Japanese product
    int N_new;
}

parameters {
    real<lower=0> GM_F; // location parameter of foreign domestic product
    real<lower=0> GM_J; // location parameter of Japanese product
    real<lower=1> GSD_F; // shape parameter of foreign domestic product
    real<lower=1> GSD_J; // shape parameter of Japanese product
    real<lower=0, upper=Y_min> RL; // Value of limit of quantification
    vector<lower=0, upper=RL>[N_cen_F] Y_cen_F; // Censored data of foreign domestic product
    vector<lower=0, upper=RL>[N_cen_J] Y_cen_J; // Censored data of Japanese product
}

model {
    RL ~ normal(0.000098, 0.0000024);
    GSD_F ~ normal(4.9, 0.8);
    GSD_J ~ normal(4.9, 0.8);
    GM_F ~ normal(8.2*10^-5, 1.4*10^-5);
    GM_J ~ normal(8.2*10^-5, 1.4*10^-5);

    Y_obs_F ~ lognormal( log(GM_F), log(GSD_F) );
    Y_cen_F ~ lognormal( log(GM_F), log(GSD_F) );
    Y_obs_J ~ lognormal( log(GM_J), log(GSD_J) );
    Y_cen_J ~ lognormal( log(GM_J), log(GSD_J) );
}

generated quantities {
    real Y_F[N_new];
    real Y_J[N_new];
    real mean_est_F;
    real mean_est_J;

    for ( i in 1:N_new) {
        Y_F[i] = lognormal_rng( log(GM_F), log(GSD_F) );
        Y_J[i] = lognormal_rng( log(GM_J), log(GSD_J) );
    }
    mean_est_F = exp( log(GM_F) + 0.5*( log(GSD_F) )^2 );
    mean_est_J = exp( log(GM_J) + 0.5*( log(GSD_J) )^2 );
}
```

Fig. S5. Stan code for estimating parameters from left-censored data assuming a lognormal distribution: comparing differences between Japanese and imported mineral water products

the difference between the groups was verified. Histograms of the generated data are shown in **Fig. S6C**. These results suggest that the probability of Cr(VI) concentrations being higher in imported MW products compared to Japanese products was 0.507.

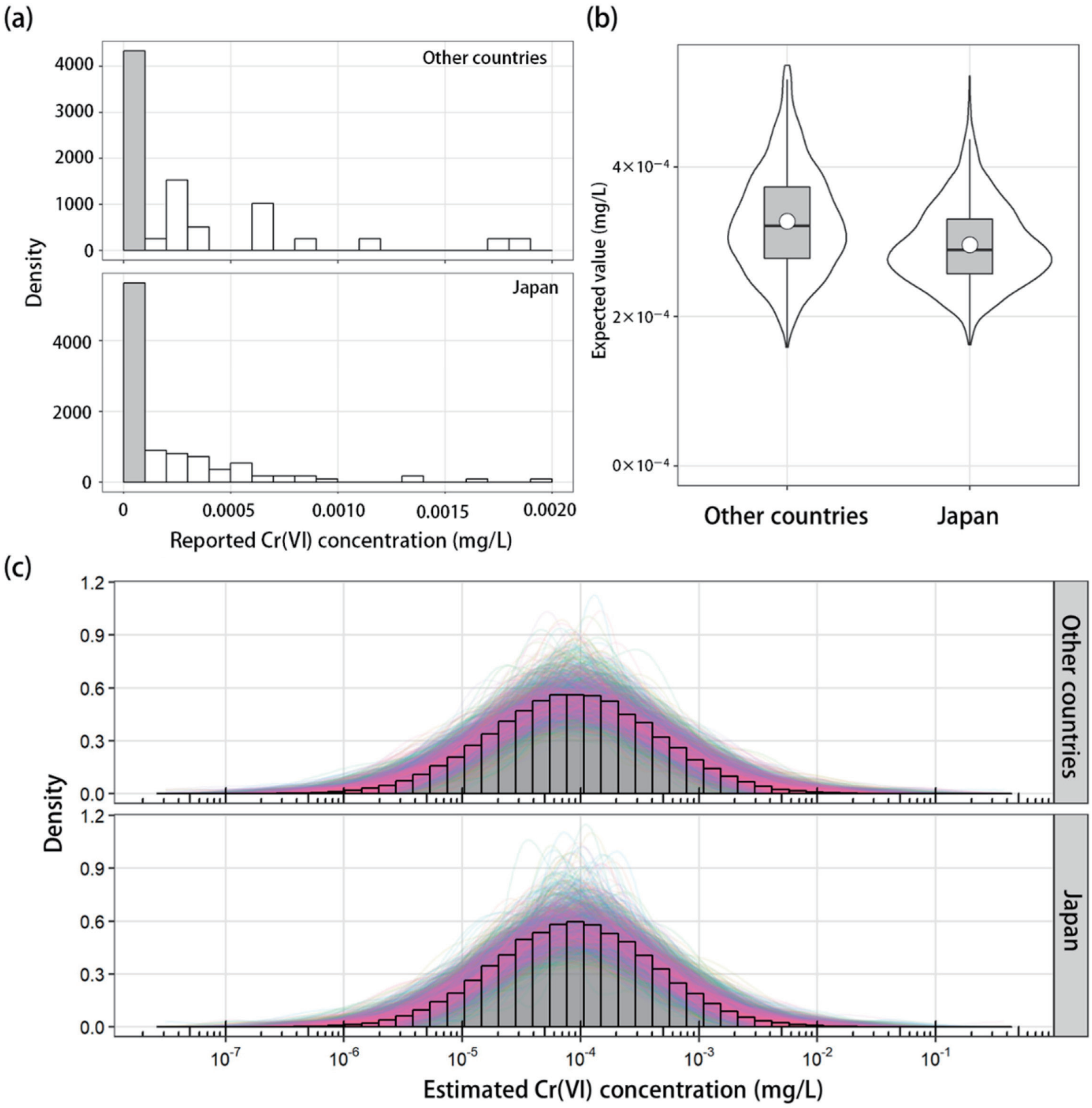

Fig. S6. Histograms of Cr(VI) concentrations in mineral water products from Japan and other countries. (a) Original data from Kataoka et al (2017). Gray bars indicate nondetects; (b) Violin plots for expected Cr(VI) concentrations in Japanese and imported mineral water products; (c) 50 random numbers generated by 2-dimensional Monte Carlo simulations from 2000 parameters estimated by MCMC. Color indicates density curves obtained from 2000 parameters.

Table S3. Group comparison of estimated parameters and summary statistics by MCMC assuming a lognormal distribution. Mean ± standard deviation and 95% credible interval are shown.

Group	GM ($\times 10^{-3}$)	GSD	Expected value ($\times 10^{-3}$ mg/L)	Predicted concentration[a] ($\times 10^{-3}$ mg/L)
Imported MW products	0.088±0.013	5.07±0.60	0.331±0.074	0.333±1.442
	0.065–0.113	3.91–6.29	0.205–0.508	0.003–2.111
Japanese MW products	0.089±0.011	4.71±0.54	0.296±0.054	0.297±1.474
	0.068–0.110	3.74–5.82	0.209–0.418	0.004–1.825

a: 50 random numbers generated by 2-dimensional Monte Carlo simulations from 2000 parameters estimated by MCMC.

References for supporting information

1. The World Health Organization. Chromium in drinking-water background document for development of WHO Guidelines for drinking-water quality. 2003. https://www.who.int/water_sanitation_health/dwq/chemicals/chromium.pdf.
2. Watanabe S. Asymptotic equivalence of Bayes cross validation and widely applicable information criterion in singular Learning Theory. *J Mach Learn Res.* 2010; **11**: 3571–3594.
3. US Environmental Protection Agency. ProUCL Version 5.1.002 Technical Guide, EPA/600/R-07/041. Office of Research and Development, Washington, DC. October. 2015.
4. Land CE. An evaluation of approximate confidence interval estimation methods for lognormal means. *Technometrics*. 1972; **14**(1): 145–158. doi:10.1080/00401706.1972.10488891
5. Helsel DR. Statistics for Censored Environmental Data Using Minitab® and R, 2nd edition. Hoboken, NJ: Wiley; 2012.
6. Lee ET, Wang JW. Statistical Methods for Survival Data Analysis. 3rd ed. Hoboken, NJ: John Wiley & Sons, Inc.; 2003.
7. Helsel DR, Cohn TA. Estimation of descriptive statistics for multiply censored water quality data. *Water Resources Research*. 1988; **24**(12): 1997–2004. doi:10.1029/WR024i012p01997
8. Gilliom RJ, Helsel DR. Estimation of distributional parameters for censored trace level water quality data: 1. Estimation Techniques. *Water Resources Research*. 1986; **22**(2): 135–146. doi:10.1029/WR022i002p00135
9. Helsel DR. More than obvious: better methods for interpreting nondetect data. *Environ Sci Technol*. 2005; **39**(20): 419A–423A. PMID:16295833, doi:10.1021/es053368a
10. Package 'EnvStats': Package for Environmental Statistics, Including US EPA Guidance. Version 2.3.12018. Millard, NY: Springer; 2013.
11. Kataoka Y, Watanabe T, Hayashi K, Ozawa R, Takizawa K, Akiyama H. Surveillance of chromium(VI) concentrations in mineral water products. *Shokuhin Eiseigaku Zasshi*. 2017; **58**(6): 275–280. doi:10.3358/shokueishi.58.275
